# Analytical Review of Geopolymer Concrete: Retrospective and Current Issues

**DOI:** 10.3390/ma16103792

**Published:** 2023-05-17

**Authors:** Besarion Meskhi, Alexey N. Beskopylny, Sergey A. Stel’makh, Evgenii M. Shcherban’, Levon R. Mailyan, Alexandr A. Shilov, Diana El’shaeva, Karolina Shilova, Memduh Karalar, Ceyhun Aksoylu, Yasin Onuralp Özkılıç

**Affiliations:** 1 Department of Life Safety and Environmental Protection, Faculty of Life Safety and Environmental Engineering, Don State Technical University, Gagarin, 1, 344003 Rostov-on-Don, Russia; spu-02@donstu.ru; 2 Department of Transport Systems, Faculty of Roads and Transport Systems, Don State Technical University, Gagarin, 1, 344003 Rostov-on-Don, Russia; 3 Department of Unique Buildings and Constructions Engineering, Don State Technical University, Gagarin Sq. 1, 344003 Rostov-on-Don, Russia; sergej.stelmax@mail.ru (S.A.S.); lrm@aaanet.ru (L.R.M.); alexandr_shilov@inbox.ru (A.A.S.); diana.elshaeva@yandex.ru (D.E.); karakarolina2001@gmail.com (K.S.); 4 Department of Engineering Geology, Bases, and Foundations, Don State Technical University, 344003 Rostov-on-Don, Russia; au-geen@mail.ru; 5 Department of Civil Engineering, Faculty of Engineering, Zonguldak Bulent Ecevit University, Zonguldak 67100, Türkiye; memduhkaralar@beun.edu.tr; 6 Department of Civil Engineering, Faculty of Engineering and Natural Sciences, Konya Technical University, Konya 42075, Türkiye; caksoylu@ktun.edu.tr; 7 Department of Civil Engineering, Faculty of Engineering, Necmettin Erbakan University, Konya 42000, Türkiye; yozkilic@erbakan.edu.tr

**Keywords:** clinker-free binder, geopolymer concrete, aluminosilicates, silica, polymerization

## Abstract

The concept of sustainable development provides for the search for environmentally friendly alternatives to traditional materials and technologies that would reduce the amount of CO_2_ emissions into the atmosphere, do not pollute the environment, and reduce energy costs and the cost of production processes. These technologies include the production of geopolymer concretes. The purpose of the study was a detailed in-depth analytical review of studies of the processes of structure formation and properties of geopolymer concretes in retrospect and the current state of the issue. Geopolymer concrete is a suitable, environmentally friendly and sustainable alternative to concrete based on ordinary Portland cement (OPC) with higher strength and deformation properties due to its more stable and denser aluminosilicate spatial microstructure. The properties and durability of geopolymer concretes depend on the composition of the mixture and the proportions of its components. A review of the mechanisms of structure formation, the main directions for the selection of compositions and processes of polymerization of geopolymer concretes has been made. The technologies of combined selection of the composition of geopolymer concrete, production of nanomodified geopolymer concrete, 3D printing of building structures from geopolymer concrete, and monitoring the state of structures using self-sensitive geopolymer concrete are considered. Geopolymer concrete with the optimal ratio of activator and binder has the best properties. Geopolymer concretes with partial replacement of OPC with aluminosilicate binder have a denser and more compact microstructure due to the formation of a large amount of calcium silicate hydrate, which provides improved strength, durability, less shrinkage, porosity and water absorption. An assessment of the potential reduction in greenhouse gas emissions from the production of geopolymer concrete compared to the production of OPC has been made. The potential of using geopolymer concretes in construction practice is assessed in detail.

## 1. Introduction

### 1.1. Relevance

The current problem of modern construction and production of building materials, products and structures is the low degree of environmental friendliness of construction processes, products, materials and structures. Thus, the world community represented by engineers, scientists, technologists, and materials scientists is aimed at obtaining environmentally friendly, highly functional materials for various industries, including construction. In this regard, it is difficult to overestimate the role of geopolymer concretes. Geopolymer concretes are a relatively new, but already well-proven way to considerably improve the ecofriendly affability and economy of structure. The thing is that these geopolymer concretes are based on the clinker-free technology of binders. In their composition, they combine industrial and other types of waste, as well as other environmentally friendly methods of production and obtaining new buildings and structures. All this gives a complex ecological and economic effect. Thus, the significance of the study is a result of the international environmental agenda and the desire of construction and production around the world to reduce resource costs: labor and material costs. The problem raised in the study consists of two aspects:

(1) Despite the rather large number of studies devoted to geopolymers, this is still a relatively new topic with a large number of promising areas for researchers. Therefore, there is a significant increase in the number of studies on geopolymer concrete, and an increase in the volume of new experimental and theoretical data about it, which need to be systematized.
(2) The practical applied research problem lies in the fact that such insufficiently deep knowledge and systematization of existing ideas do not allow in some way to unify and standardize certain approaches, technologies and compositions, although this could in principle be done to improve understanding in the world community of efficiency and correct use of geopolymer concretes and geopolymer technologies.

### 1.2. Background

**Figure 1:**
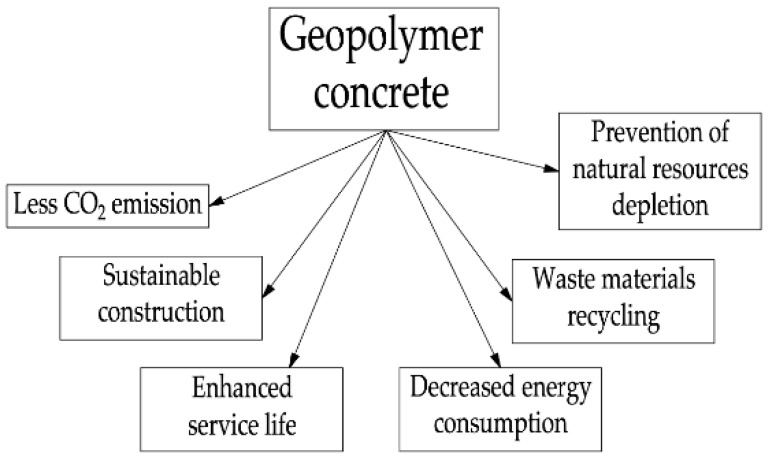
Main advantages of geopolymer concretes.

Concrete based on ordinary Portland cement (OPC) is one of the main structural resources of contemporary construction [1]. High strength properties, wide availability and relatively low cost make it a versatile material for an extensive range of tasks in building manufacturing. However, the production of OPC is associated with a large amount of energy, natural raw materials (limestone, fossil fuels), and the production of cement clinker is associated with significant greenhouse gas emissions into the atmosphere. According to [2,3,4], for every ton of cement clinker manufactured, there are 0.8 tons of CO_2_, the emissions of which are the main cause of global warming, along with deforestation and burning of fossil fuels [5]. This disadvantage of OPC is not sustainable and, despite its outstanding properties, makes it urgent to find more environmentally friendly and cleaner alternatives. This is especially important given the latest research for 2022, which suggests that the amount of CO_2_ in the atmosphere has reached 421 parts per million (ppm), while the norm for clean air is only 300 ppm. This is a record value that exceeded the values of previous years (419 ppm for 2021 and 417 ppm for 2020) [6]. Several studies have been devoted to solving a similar problem—the disposal of agricultural, industrial waste, fuel, and energy complex in concrete, but at the same time in concrete based on cement [7,8,9,10,11,12,13,14,15,16,17,18]. At the same time, it should be noted that geopolymer concretes are even more promising, environmentally friendly, and economical due to the absence of clinker binders in them and the use of more environmentally friendly cheap components in their composition, which can significantly approach the implementation of the global environmental agenda. One of the main environmentally friendly alternatives to OPC are geopolymers due to their smaller carbon footprint [19]. Figure 1 shows the main advantages of geopolymer concretes.

Initially, the concept of structure creation of geopolymer resources was offered in 1979 by the French investigator J. Davidovits [20,21,22,23,24,25]. Despite the fact that initially, they did not become the subject of wide interest of scientists and researchers, in the last 12 years there has been an exponential rise in the amount of publications devoted to various aspects of the study of geopolymer materials and various technologies for their production. Interest in geopolymer materials is due to their technological and operational characteristics [26]. Long service life, low carbon emissions, high strength, and durability, as well as chemical inertness with respect to many solvents and aggressive environments, together with resistance to high temperatures, make geopolymer materials extremely promising for use not only in the construction industry, but also in medicine, and in industrial production [27,28]. The term “geopolymers” itself is named so due to the fact that the raw materials for the manufacture of these materials are minerals of geological origin. The first geopolymer material was the product of the interaction of components containing aluminates and silicates in an alkaline environment [20]. Later, the concept of environmentally friendly geopolymer concrete was developed by Erez N. Allouche [29], a researcher from the USA who presented the basic formula for geopolymer compositions using components of exclusively natural origin in its development. The growing popularity of the use of geopolymer materials will have a beneficial effect on the environment by reducing CO_2_ emissions into the atmosphere, which is consistent with the concept of sustainable development [30]. Complete replacement of OPC with a geopolymer analog based on aluminosilicates will reduce CO_2_ emissions into the atmosphere by 80%. However, due to the lack of standard regulations and a number of disadvantages such as high construction costs, high shrinkage and a fast-curing process, geopolymer materials are currently rarely used compared to OPC. In the early stages of the development of the technology of geopolymer materials, rather expensive materials with high-performance characteristics were created for aviation, automotive and other productions. Future geopolymerization procedures started to be used for the synthesis of relatively economical construction resources established on thermal power plant ash, slag, waste from mining and handling of rocks, and other manufacturing waste, which partially solves the problem of the high cost of such compositions [31].

Despite the absence of standard regulatory documents on geopolymer materials, at the moment a fairly large amount of research has been accumulated that considers their properties and production technologies. Comprehensive systematic reviews of the technology of the compositions and technical applications of geopolymer concrete are known, including environmental and economic aspects [32,33,34,35,36]. The increased interest of researchers in the subject of geopolymer concretes over the past 12 years has led to the fact that today not only important studies are available, but also reviews on research related to geopolymer concretes. Summarizing the above, it is necessary to emphasize the importance of a critical comparative analysis of previously published works with this study. The review [37] is devoted only to the mechanical properties of geopolymer concretes in the fresh and hardened state, as well as their durability. The review [38] covers the microstructural properties of geopolymer paste, as well as its strength characteristics. The reviews [39,40] were devoted to the physical and mechanical characteristics of geopolymer concretes based on ground granulated blast-furnace slag (GGBS) and fly ash (FA). The studies [41,42] were devoted to the effect of various mineral additives on the physical and mechanical properties and microstructure of geopolymer concretes. In addition, reviews of data on the selection of compositions and proportions of mixture components for geopolymer concretes are known. Various precursors and activators used in geopolymer concretes have been carefully considered in [43,44,45,46,47]. It is important to note that one of the main goals of the above studies was to find mortars for the implementation of the concept of sustainable development in the field of finding environmentally cleaner and less energy-intensive alternatives to OPC.

It should be noted that in the open press at the moment there are quite a few reviews containing an analysis of data on the selection of compositions and proportions of mixture components for geopolymer concretes based on various types of binder [28,32,33,34,35,36,43,44,45,46,47,48,49,50,51,52,53]. This review presents a comprehensive study of the components of a geopolymer concrete mix, as well as a critical review of the various mix selection techniques used for geopolymer concretes, considering certain input and output variables. In addition, attention is paid to studies published in recent years on the physical-mechanical and microstructural properties of geopolymer concretes, as well as their durability. The main factors influencing the properties of geopolymer concrete at all stages of hardening are proposed. The study of nanomodified geopolymer concretes, 3D printing using geopolymer concrete, geopolymer concrete reinforced with steel bars, and assessment of the impact of geopolymer concrete technology on global warming potential are considered.

### 1.3. Rationale

Thus, the study is intended to solve some problems of systematization of existing ideas and develop an empirical and literary base for future research and point strengthening of the world theory of geopolymer concretes. The purpose of the study is a detailed in-depth analytical review of studies of the processes of structure formation and properties of geopolymer concretes in retrospect and the current state of the issue. To achieve this goal, the present study solves the following tasks:

(1) The main prescription, technological, constructive, engineering and scientific approaches to solving the problems of the best and most ecological and economic efficiency of geopolymer concrete for various types of climatic zones, regions, buildings, structures, and various levels of their responsibility are determined.
(2) The main factors and main criteria influencing the final quality and efficiency of geopolymer concretes are identified.
(3) The main fundamental relationships among the composition, construction and properties of geopolymer concretes are determined.
(4) Interrelation at micro- and macrolevels was revealed in the formation of the construction and properties of geopolymer concretes.
(5) The factors of raw materials and the correct dosage of the component composition were evaluated.

Dependences between such compositions and dosages and output parameters in the form of properties of geopolymer concretes and the final reliability of buildings and structures are revealed. An assessment is given both from a quantitative and a qualitative point of view for all relationships between geopolymer concretes, their compositions, properties and final energy, resource and material efficiency.

This article provides a global overview of the state of the art in current research on geopolymer materials. Section 2 is devoted to a detailed analysis of the components of geopolymer materials and their ratios, as well as the process of geopolymerization itself. Section 3 discusses the main physical and mechanical properties of fresh and hardened geopolymer concretes, as well as the main factors affecting them to one degree or another, as well as the durability of geopolymer concretes. Section 4 discusses the effect of adding OPC to geopolymer concrete. Section 5 provides an overview of recent innovations in geopolymer concrete such as 3D printing, global warming assessment, nano-modified and self-sensing geopolymer concrete. The main conclusions are given in Section 6.

### 1.4. Methods

**Figure 2:**
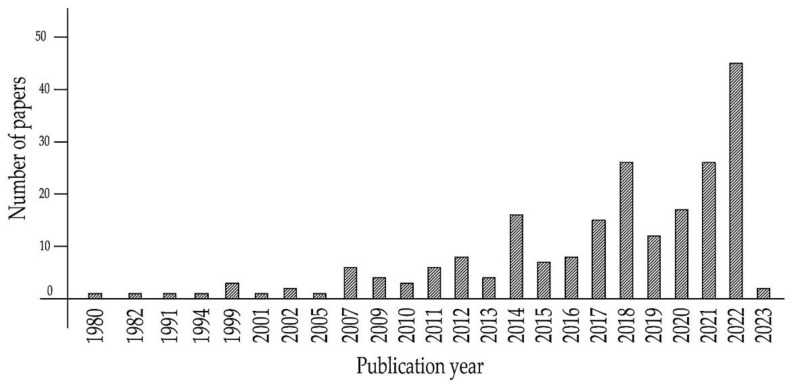
Timeline of the literature used by the authors.

The study was carried out using methods including a review, world-class research on the topic of geopolymer concretes in databases of international journals in open and closed access, the study of patented technologies, and the study of the raw material base, in relation to each region of the world. Criteria such as journal, year of publication, keywords, and relevance of the material to the subject of this review article were used to determine whether the material relates to this review article. The most frequently considered parameters of geopolymer concretes in scientific articles are compressive strength (CS), tensile strength (TS), flexural TS, modulus of elasticity, hardening time, workability, shrinkage and structural changes that occur during material hardening. The initial data were information about the compositions, recipes, technology, and properties of various geopolymer concretes created and used in various regions of the world. The fundamental interactions occurring at the micro- and macrolevels in geopolymer concretes of various types were studied. As an accounting and analysis of risks in the course of the research, a subject area was identified from those sources that have similar features to each other, thereby allowing some verification of the results of some authors based on the results of other authors. Visualization methods, flowcharts, photographic materials, and tabular methods are used, thus the clarity of the study demonstrates the scientific novelty and analytical aspect of the review study. The timeline of the literature used by the authors is shown in Figure 2.

## 2. Geopolymer Concrete as an Environmentally Friendly Composite Material

### 2.1. Structure Formation Mechanism of Geopolymer Binders

Geopolymer concrete is a building material that is an inorganic polymer made by thermally activating natural materials with a high content of silica and alumina with alkaline mortars that polymerize these materials into molecular chains that create the structure of a hardened binder. As a rule, the raw materials are by-products of agriculture and heavy industry, which must be disposed of, for example, kaolinite, bentonite, FA, rice husk ash, and wheat straw ash.

Despite the similarity of geopolymer materials with traditional materials of alkaline activation, there are main differences between them, consisting of different chemical compositions, due to the structure formation of the material during the activation process. Geopolymer materials form a stable spatial polymer structure during polymerization, while conventional alkali-activated materials form unstable monomers during activation. As a result, geopolymer materials have lower strength than alkali-activated materials, but compared to the latter, they have much greater durability [54]. The validity of the term “Geopolymer materials” is based on the fact that the technology of geopolymer binders provides for the synthesis of the polymer structure of such materials from monomeric silicate and aluminate groups that can be formed during the destruction of the primary structure of rocks or industrial wastes of aluminosilicate composition in alkaline solutions. Thus, according to the authors of [54], an alkali-activated binder is a geopolymer only if it is a material consisting of amorphous to semi-crystalline zeolite.

Geopolymer concrete can be made from both one-component and two-component compositions. Traditionally, geopolymer concretes were presented precisely in the form of two-component compositions, in which solid aluminosilicate raw materials for preparation for work must be mixed with a liquid alkaline activator composition [55]. One-component compositions simplify transportation and work with geopolymer concrete and are a dry mixture of aluminosilicate raw materials and alkaline activators in solid form, the preparation of which requires mixing with water [48].

Despite the huge amount of research on the mechanism of structure formation of clinker-free binders of alkaline activation, at the moment it remains not fully understood. The complexity of studying the mechanism of hardening of geopolymer binders is primarily determined by the fact that production wastes are used in their manufacture, chemical composition, and physical properties, which are not constant [56,57,58,59].

The structure formation of geopolymers based on FA can be divided into three stages. In the first stage, the FA aluminosilicate is dissolved and hydrolyzed to form aluminate and silicate monomers. In the second stage, aluminum and silicon ions, which are converted into oligomers, form a gel with rather large networks as a result of condensation. In the third stage, the gel continues to restructure and an amorphous structure develops in the form of an aluminosilicate network due to the polycondensation reaction [40].

Of the entire set of geopolymer binder systems, one of the most studied mechanisms of structure formation are systems based on GGBS. The Si–O–Si and Al–O–Si compounds in the medium of a highly concentrated mortar undergo destruction and pass into a colloidal state; then, with an increase in the number of colloidal particles, they are compacted and strengthened [60,61]. Thus, the hydration products of the GGBS-based geopolymer binder are calcium hydrosilicates and sodium hydroaluminosilicates. Additionally, in the presence of clay minerals in the binder system, hydroaluminosilicates are formed [62]. In turn, experiments performed using X-ray diffraction and differential thermal analysis, scanning electron microscopy in combination with X-ray microanalysis confirm this statement that calcium hydrosilicate gel is the hydration product of geopolymer binder systems based on GGBS [63].

**Figure 3:**
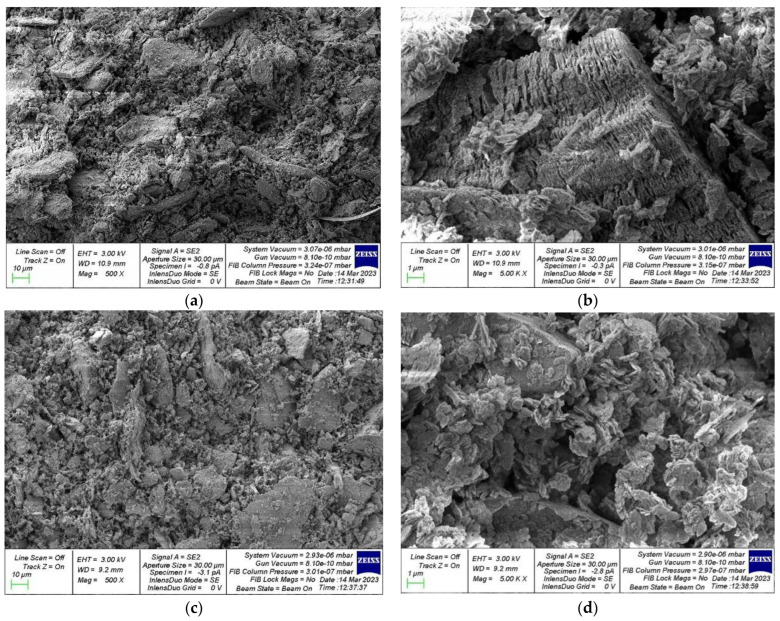
Photographs of the microstructure of geopolymer concrete based on fly ash (**a**,**b**) and ground granulated blast-furnace slag (**c**,**d**) with magnifications of 500× (**a**,**c**) and 5000× (**b**,**d**).

Figure 3 shows photographs of the microstructure of geopolymer concrete based on FA and GGBS.

Let us analyze our SEM analysis during the study of geopolymer concretes based on FA (Figure 3a,b) and GGBS (Figure 3c,d). Ash-based geopolymer concrete has a more coherent structure, and denser packing of particles, which is facilitated by better interaction between the components inside the geopolymer concrete, and during the physical and chemical processes of hardening structure is more developed. There is a smaller number of micropores and voids, which ultimately contributes to a more perfect dense packing of particles, and good contact of various components at the level and phase boundaries, which ultimately contributes to the formation of high physical and mechanical characteristics and high performance of geopolymer concrete on FA.

Geopolymer concrete on GGBS also has a characteristic structure, in principle confirming its high suitability as a component for geopolymer concrete; however, the structure is somewhat different. There is a large degree of coarseness of the created structure, a granular structural characteristic of the material, and a more pronounced boundary of various components and phases inside the concrete. The packing of particles also has some voids and micropores, which indicates some advantages of geopolymer concrete on FA over geopolymer concrete on GGBS.

At the same time, experimental studies and pilot testing have proven the applicability of both types of geopolymer concrete in practice with some assumptions and reservations. In general, this SEM analysis is in good agreement with the experimental studies performed and the literature data of other authors.

### 2.2. Main Raw Materials of Geopolymer Composite Binder Systems

**Figure 4:**
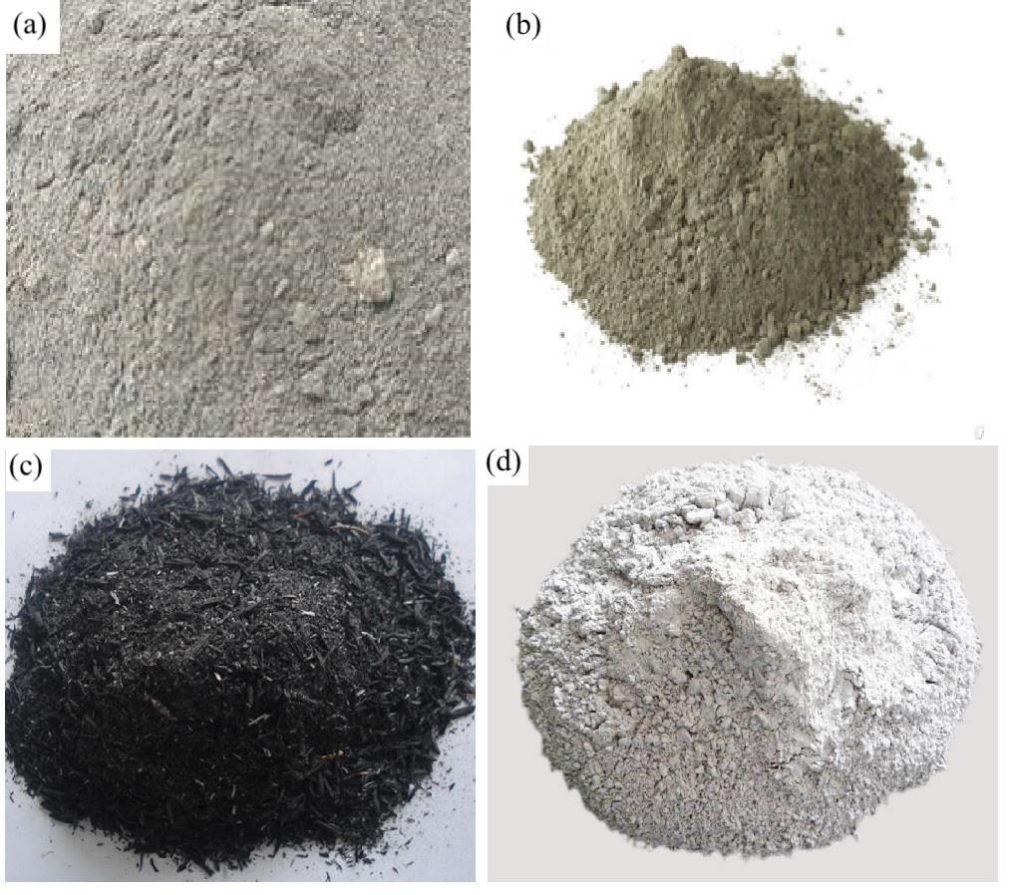
The main forms of aluminosilicate raw materials used in the technology of geopolymer concretes (**a**) fly ash, (**b**) metakaolin, (**c**) rice husk ash, (**d**) ground granulated blast furnace slag.

Geopolymer binder systems are a mixture of an aluminosilicate component and an alkaline activator. It should also be noted that this binder system can include one or several types of aluminosilicate components and alkaline activators [19]. The main types of aluminosilicate raw materials applicable in the technology of geopolymer concretes are shown in Figure 4.

The most used in practice are geopolymer concretes based on FA, metakaolin, and GGBS [28]. As a rule, they all have different physical and strength characteristics, which directly depend on the content of aluminum and silicon oxides in the binder component used in their manufacture. Table 1 provides an overview of the different types of aluminosilicate components used in geopolymer concrete technology, with their chemical composition.

In the studies presented in Table 1, the authors used such aluminosilicate components as FA, metakaolin and GGBS. From the presented analysis, it was established that the content of silicon and aluminum oxides in FA varies, respectively, between 25 and 73% and 21 and 34%, in metakaolin 48 and 59% and 34 and 37%, in rice husk ash 83 and 90%, in hammered GGBS 30 and 42% and 8 and 35%.

To activate the hardening processes of binders by alkaline activation, sodium hydroxide, potassium hydroxide, sodium, potassium, potassium-sodium liquid glass, and soda ash are used. The geopolymerization reaction depends on the concentration of alkaline mortars and their reactivity. Thus, when developing various geopolymer systems, it is important to correctly determine these characteristics of mortars. A list of various alkaline activators and their characteristics based on the results of several studies is presented in Table 2.

According to the results of studies [110,111,112,113,114,115], it can be concluded that the type and composition of the activator are the determining factors affecting the values of strength characteristics. As can be seen from Table 2, the most commonly used activators in geopolymer composites are Na_2_SiO_3_ and NaOH, the hydroxide molarity varies from 6 to 14 M. Geopolymer composites containing only hydroxide in their composition are characterized by low strength values, a large number of pores and shrinkage cracks, and the inclusion of a source of silicate leads to an acceleration of the hardening process [116,117].

### 2.3. Selection of Compositions of Geopolymer Concretes

As with concrete in OPC as a binder, geopolymer concretes also require careful and rational selection of its components and their ratios. Despite the fact that the characteristics of concretes based on OPC mainly depend on the proportion of the components of the mixture, for geopolymer concretes, the selection of the composition is complicated by the fact that its properties are significantly influenced by a larger number of factors. For example, curing time and temperature, the proportion of water and solid components, alkali content, type and composition of aluminosilicate raw materials, and the proportion of components involved in polymerization (aluminates and silicates, silicates and hydroxides) [118,119,120,121,122,123]. The selection of compositions, as in the case of conventional concrete with Portland cement as a binder, can be carried out based on several methods. The mathematical method of trial and error is popular, based on the parameters of the strength and fluidity of the composition. In this method, the required values for CS and slump are first established, then the binder content and alkali-to-binder proportion values are selected; a preliminary calculation of the required amount of coarse aggregate is performed and the amount of alkali activator is calculated. This method was developed by several researchers who, based on the trial-and-error method, built an algorithm that took into account a larger number of factors, for example, the proportion of activator to binder, silicates to hydroxides, etc. The main goal of this method is to design the most durable geopolymer composition, considering the influence of various mixture parameters [124]. The method based on the final strength of the composition implies that only the main decisive factors affecting the strength are considered, for example, the proportion of water to binder, or activator to binder. The selection of the composition of the mixture is carried out based on the results of tests for strength and workability, presented in the form of graphs, which additionally indicate parameters such as the proportion of water to the binder, activator to binder, silicates and hydroxides, activator concentration, aggregate size, temperature, curing time, composition silicates, etc. This method has also been developed in the form of new approaches to the selection of composition based on strength. In particular, as a basis for selecting the composition, instead of the graphs indicated above, it is proposed to use a graph based on four parameters: the proportion of water to binder, activator to binder, silicates to hydroxides and activator concentration. Additionally, the selection of the composition based on strength, proposed in the ACI standard, considers the influence of the proportion of activator and binder, the concentration of the activator and the particle size modulus of the aggregate [125]. The composition selection method based on the proportion of the activator to the binder suggests selecting parameters such as the hydroxide concentration and the proportion of silicates to hydroxides from the literature [126,127,128,129]. The content of the activator is proposed to be set equal to 200 kg/m^3^. Further, it is proposed to determine the strength or proportion of activator to binder based on experimental data. The method of composition selection based on the proportion of binder to sand involves the initial determination of the optimal proportion of binder to sand [130,131]. Next, the concentration of hydroxides and the optimal proportion of silicates to hydroxides are calculated. After that, the concentration of the activator and the ratios of the activator to the binder and water to the binder are established, considering the dependence of these parameters on each other.

### 2.4. Polymerization Process of Geopolymer Concretes

**Figure 5:**
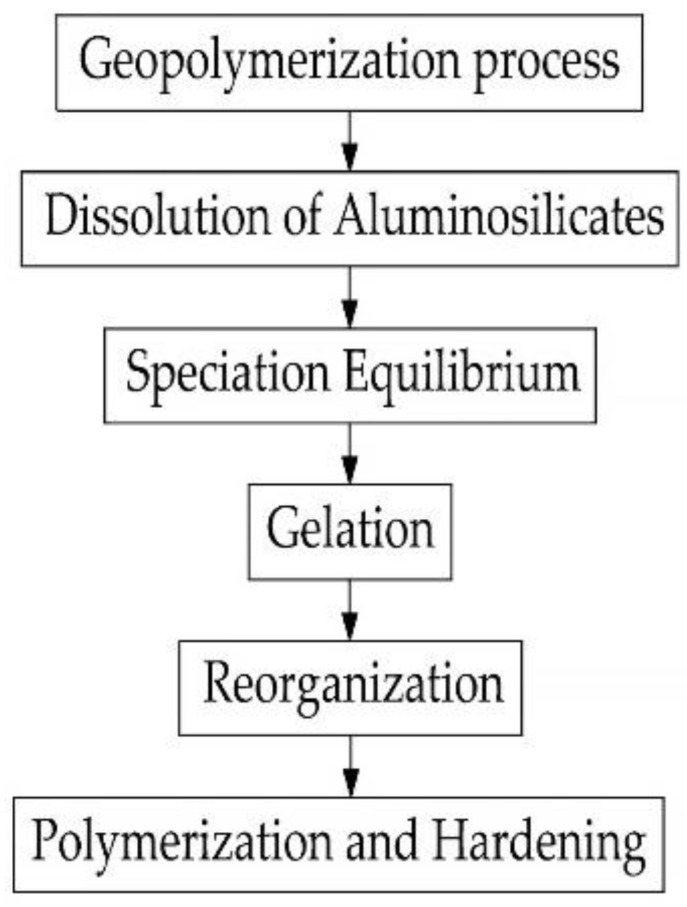
The main stages of the process of polymerization of geopolymer compositions.

The process of polymerization of geopolymer compositions implies the formation of a spatial polymeric aluminosilicate network during the rapid chemical reaction of silica and aluminum oxides under the action of an alkali-containing activator mortar. Particles of silica and aluminum oxide are dissolved in the alkaline medium of the activator mortar with subsequent transformation into a spatial chain of the aluminum silicate polymer structure. The type of geopolymer depends on the composition of aluminosilicates [132]. The properties of geopolymer concrete are formed based on the binding of calcium silicate hydrate and the polymerization process. In geopolymer concretes with the complete replacement of OPC with aluminosilicates, the polymerization process occurs during the activation of aluminosilicates, during which aluminosilicates are oxidized in an alkaline medium of an activator mortar with the further dissolution of the resulting aluminosilicate oxides in a mortar with a high pH, resulting in the formation of a gel consisting of oligomers based on Si-O-Si and Si-O-Al polymer bonds, ultimately forming the structure of the geopolymer composition; when hardened, it forms a polymer structure that retains aggregates and the unreacted particles of the composition. In geopolymer concretes with the complete replacement of OPC by aluminosilicates, instead of the formation of calcium silicate hydrate gel, polymers are synthesized from silica and alumina particles [133]. Figure 5 shows the main steps in the polymerization process of geopolymer concretes.

The most studied and fully presented in the literature is the structure formation of a slag-alkaline binder based on GGBS. As mentioned earlier, in a high-concentration alkaline solution, the Si-O-Si and Al-O-Si compounds dissolve and pass into a colloidal state. After an increase in the number of colloidal particles, they are compacted and hardened. In [134], it is said that the activation mechanism is a series of successive destruction-densification reactions, which result in the destruction of the structure of raw materials and their transformation into low-stability structural units that interact with coagulation structures and subsequently become compacted. In the first stage, the destruction of the Si-O-Si and Al-O-Si compounds in a highly concentrated alkaline solution and their transition to a colloidal state occurs. Further, in the second stage, there is an increase in the number of colloidal particles; in the third stage, their compaction in the existing volume causes the process of autogenous shrinkage [134]. The hydration products of the slag-alkaline binder are formed as a result of the interaction of calcium hydrosilicates and sodium hydroaluminosilicates. Additionally, in the presence of clay minerals in the binder as a result of their interaction with an alkaline activator, hydro-aluminosilicates (zeolites) are synthesized. In [135] it was suggested that zeolite phases (tobermorite, hydroxosodalite) and crystalline compounds Na_2_O-Al_2_O_3_-SiO_2_-H_2_O and Na_2_O-CaO-Al_2_O_3_-SiO_2_-H_2_O are formed only at a high proportion of water to binder. In the course of studies carried out by X-ray diffraction analysis, it was found that at a high rate of slag hydration, CSH gel (nCaO SiO_2_ mH_2_O) is formed, and at later stages of hydration, hydrotalcite is formed [134]. According to the authors of [136], the products of the reaction of hydration of the slag binder are calcium hydrosilicates and xonolite. In the course of experimental studies performed by the methods of X-ray diffraction and differential thermal analysis of scanning electron microscopy in combination with X-ray microanalysis, it was confirmed that the product of hydration of a geopolymer binder based on GGBS is calcium hydrosilicate gel at a low C/S proportion [137]. Using the same method, the authors of [136] found that there are no crystalline reaction products in the products of alkali-activated slag hydration while using electron microscopy methods a month after the start of hydration, hydrotalcite, calcite (CaCO_3_) and calcium hydrosilicate.

**Figure 6:**
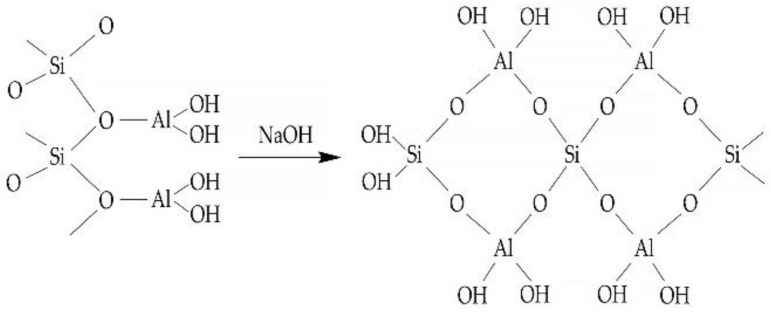
Scheme of polycondensation of kaolinite SiO_2_, Al_2_(OH)_4_ in an alkaline medium [138].

The first material on the basis of which a geopolymer composition was obtained was heat-treated kaolin, which hardened under the influence of alkali. J. Davidovits [138] proposed a scheme of the polycondensation process that occurs during the hardening of the geopolymer, shown in Figure 6.

**Figure 7:**
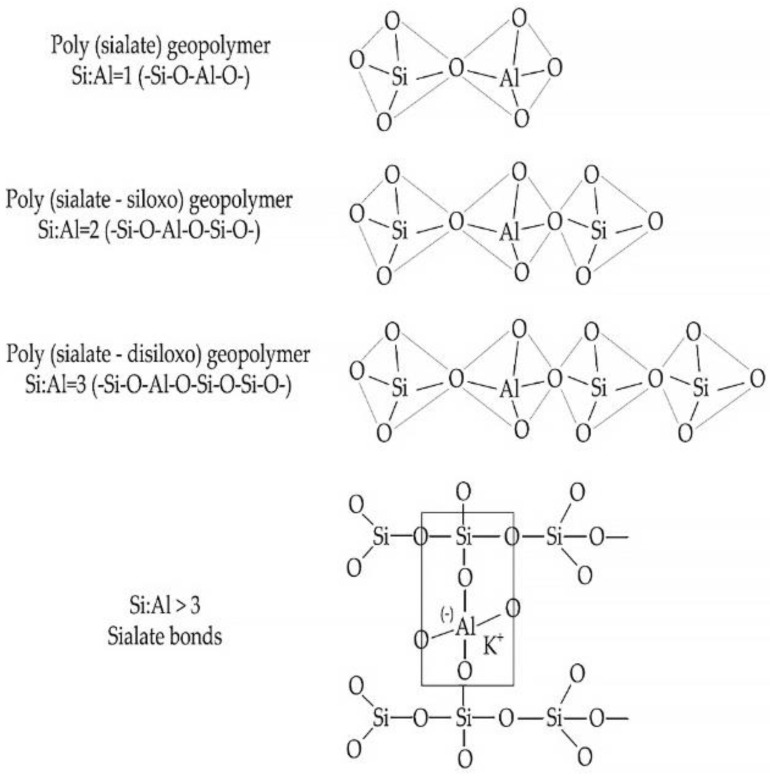
Structural scheme of geopolymer compounds [139,140,141,142,143,144].

Proceeding from these concepts, as well as from the concepts presented in works [139,140,141,142,143,144], geopolymers are polymeric materials, since they have a structure with silicon and aluminum atoms repeating in chains. Depending on the alternation of these atoms, geopolymer materials can be subdivided into silates, polysilato-siloxes, and polysilato-siloxo-(disiloxo). The block diagram of these geopolymer compounds is shown in Figure 7.

In [139] author considers that the geopolymerization reaction proceeds in three stages:

- In the first stage, silicon and aluminum oxides are dissolved in an alkaline medium of a concentrated solution of NaOH or KOH;
- in the second stage, natural polymer structures are split into monomers;
- in the third stage, setting and compaction occurs as a result of the conversion of monomers into polymeric materials.

**Figure 8:**
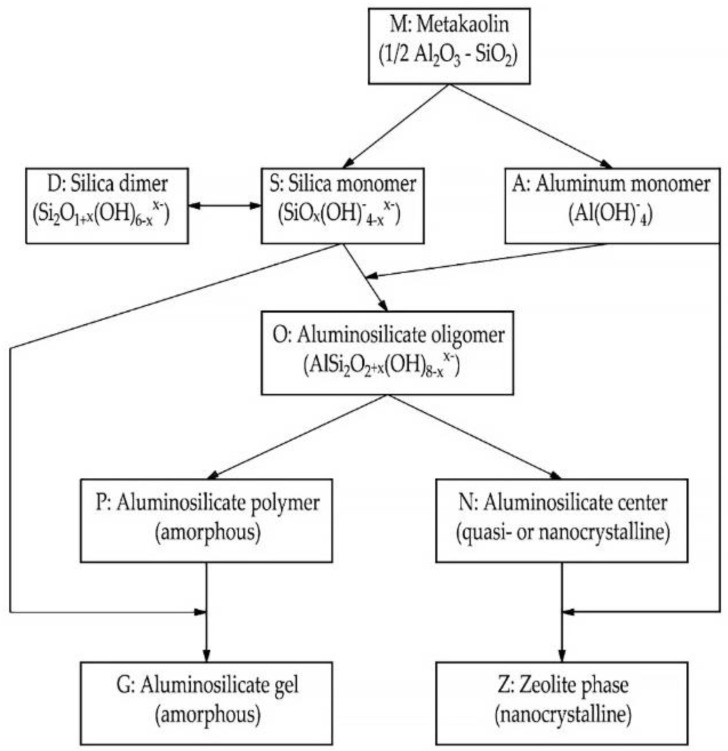
Stages of the geopolymerization process according to the model [147].

In the hardening geopolymer compositions, spatial aluminosilicate structures with the empirical formula M {-(Si-O)z-Al-O}n·w·H_2_O are gradually formed, where M is the atoms or cations of K, Na or Ca; n is the degree of polycondensation; z is 1, 2, 3 or more. The structure of the material is formed by [SiO_4_]^4−^ and [AlO_4_]^5−^ tetrahedra, interconnected by oxygen bridges. Si-O-Al compounds are closed in chains and rings. Positive ions (Na^+^, K^+^, Ca^2+^) compensate for the negatively charged four-coordinate Al. In the course of the studies conducted using the methods of thermal analysis, nuclear magnetic resonance, and mercury porosimetry, it was found that the pores of geopolymer compositions contain water and sodium or potassium cations that have not entered into a chemical reaction with the binder. When dried, they migrate to the surface of the material and undergo atmospheric carbonization, which is the cause of efflorescence on the surface of products made of geopolymer materials [139]. According to [145] the dissolution of vitreous aluminosilicate at the first stage of geopolymer structure formation proceeds as follows: first, H+ ions are exchanged for Ca^+^ and Na^+^, then hydrolysis of aluminosilicate compounds, destruction of the depolymerized glassy structure, and splitting of Si and Al compounds into unstable monomeric structures [145]. According to a simplified model proposed by the authors of the study [146], the geopolymerization process begins with the dissolution of finely ground thermally treated aluminosilicate raw materials in an alkaline medium. At the same time, the degree of dissolution of the aluminosilicate raw material is affected by its dispersion and the reactivity of aluminum in the raw material. The dissolution of aluminosilicate raw materials by alkaline hydrolysis occurs with a sufficient amount of water and is accompanied by the destruction of aluminum and silicon compounds, which go into solution and accumulate in the form of individual particles on the surface (in monomeric form). As solid particles accumulate in the solution, its polymerization occurs, the so-called geopolymerization [146]. Researchers [147] proposed a more complex model of the chemical processes of hardening geopolymers. According to it, it is believed that after several stages of transformation, an amorphous aluminosilicate gel and a zeolite phase are formed from the silicate and aluminate monomers. That is, during hydration, with an increase in the solubility of aluminum K/Al in an alkaline solution, a decrease in the Si/Al proportion occurs. The stages of the geopolymerization process, according to the model [147], are shown in Figure 8.

The synthesis of geopolymer materials based on aluminosilicate raw materials with a high content of Al_2_O_3_ nanoparticles activated with alkaline hydroxide occurs through surface phase separation without passing through the induction period inherent in binders with such an activator [148]. At the initial stage of the reaction, the geopolymer gel predominates over the silica gel. As the mixture solidifies, a zeolite phase is formed, which contains crystalline faujasite, and the structure of the Na-F geopolymer becomes of the edingtonite type. Subsequently, such an explanation of the mechanism of geopolymerization through alkaline dissolution was widely disseminated in scientific publications. In the synthesis of geopolymers, the process of converting solid particles into a gel does not occur in a highly alkaline environment, but under poorly solvated conditions. At the first stages of the creation of geopolymer materials, they included only materials obtained based on metakaolin in the study of alkaline binders; it was concluded that similar hardening mechanisms are also characteristic of similar materials based on FA, GGBS, heat-treated feldspar rocks, as well as other rocks and technogenic products of aluminosilicate composition.

**Figure 9:**
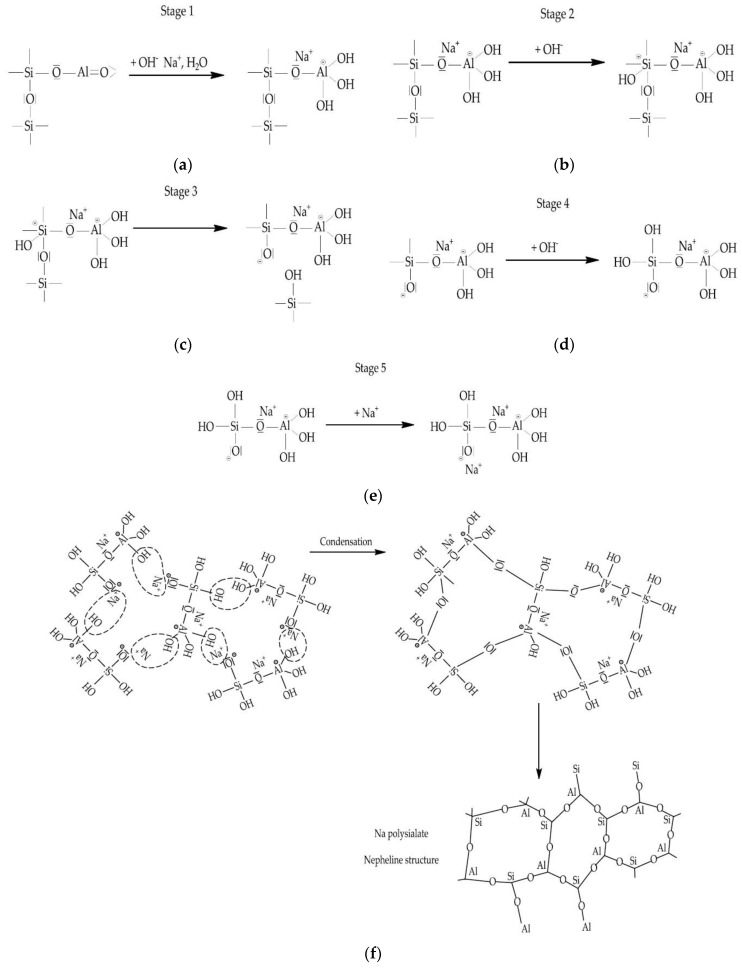

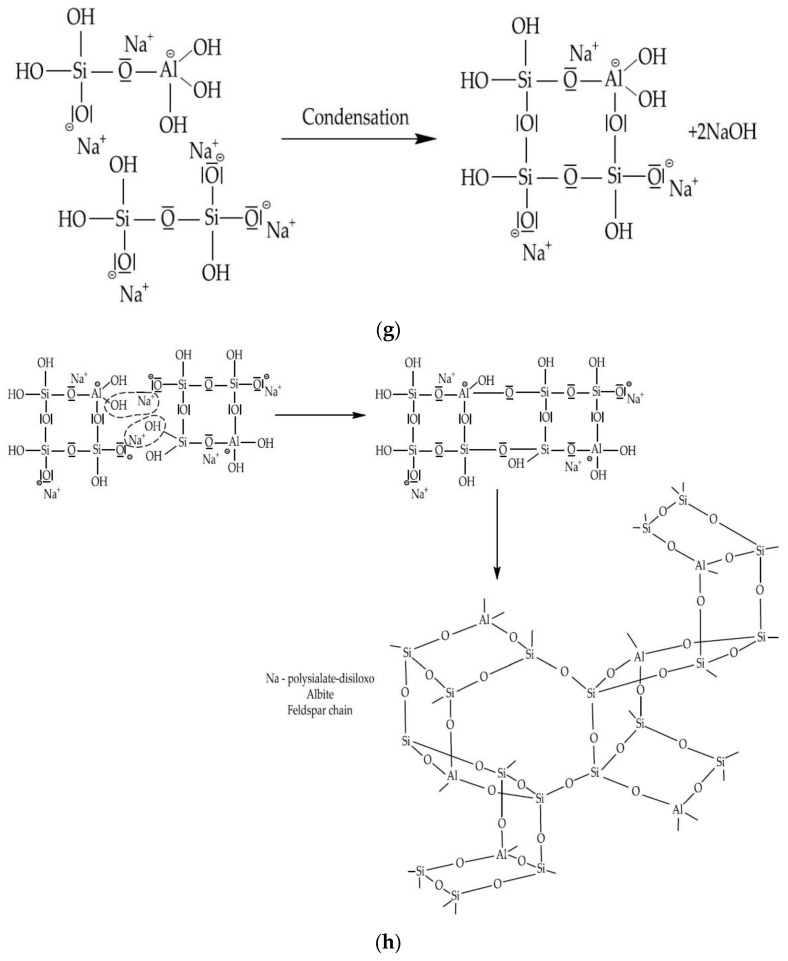
Stages of the geopolymerization process according to the model of [140]: (**a**) stage 1; (**b**) stage 2; (**c**) stage 3; (**d**) stage 4; (**e**) stage 5; (**f**) stage 6 in the case when NaOH acts as an activator; (**g**) stage 6 in the case when liquid glass acts as an activator; (**h**) stage 7.

Work [140] based on the analysis of studies of the process of hardening of geopolymer materials describes the mechanism of their structure formation using chemical reactions through interaction with NaOH or KOH and divides it into the following stages (Figure 9).

At the first stage in Figure 9a during the initial interaction with an alkaline activator, tetravalent Al is formed in the side group of silates -Si-O-Al-(OH)^3−^Na^+^.

At the second stage in Figure 9b, the dissolution of alkali begins with the addition of hydroxyl groups OH^−^ to silicon atoms, as a result of which the valence of electrons increases to a five-covalent state.

At the third stage in Figure 9c, the oxygen contained in the -Si-O-Si siloxane is split off by electron transfer from Si to O with the formation of intermediate silane groups -Si-JH and basic siloxo groups Si-O-.

At the fourth stage in Figure 9d, the formation of Si-OH silane groups continues, and orthosilates are formed, which are the primary nuclei of geopolymers.

At the fifth stage in Figure 9e, the main Si-O- compounds interact with sodium cations Na^+^, resulting in the formation of simple (terminal) Si-O-Na bonds.

At the sixth stage when NaOH is used as an activator as in Figure 9f, condensation occurs between orthosilate molecules, reactive Si-O-Na groups and OH-Al- hydroxoaluminate groups to obtain NaOH and form cyclotrisilate structures, thus NaOH alkali is released and again enters into polycondensation reaction with the formation of sodium polysilate nepheline structures. If liquid glass (soluble Na-polysilate) is used as an activator at this stage in Figure 9g during the condensation of disilicate and orthosilicate molecules of the reactive groups Si-O-Na, Si-OH and hydroxoaluminate groups OH-Al-, an orthosilate-disiloxic cyclic structure is formed, where alkali NaOH is released and reacts again.

At the seventh stage in Figure 9h, the polycondensation of the albite structure of the sodium polysilicate-disiloxo continues with the formation of typical feldspar chain structures [60,61].

The mechanism of chemical reactions that take place during structure formation is the basis for the study of geopolymer materials, in particular, their properties, structure formation features, types of alkaline binders and mixture formulations. In conjunction with the fact that the scope of geopolymer concretes, their structure and properties depend on the Si/Al ratio [60,61], understanding the essence and processes of geopolymerization, as well as knowledge of the mechanism of chemical reactions occurring in this case is the most important aspect in designing geopolymer materials, products and structures from them, as well as creating new composites.

## 3. Physical and Mechanical Properties of Geopolymer Concretes

When considering geopolymer concrete as an alternative to concrete with a binder in the form of OPC, it is necessary to consider the properties not only of fully cured concrete but also of the ready-to-lay mixture. Fresh mix properties include workability, setting time, shrinkage and structural changes during the setting process. It is important to note that the process of polymerization of geopolymer compositions is exothermic, so the temperature released by geopolymer concrete during hydration is also an important parameter. The properties of fully cured concrete include CS, axial TS, flexural TS, and modulus of elasticity [50,142].

**Figure 10:**
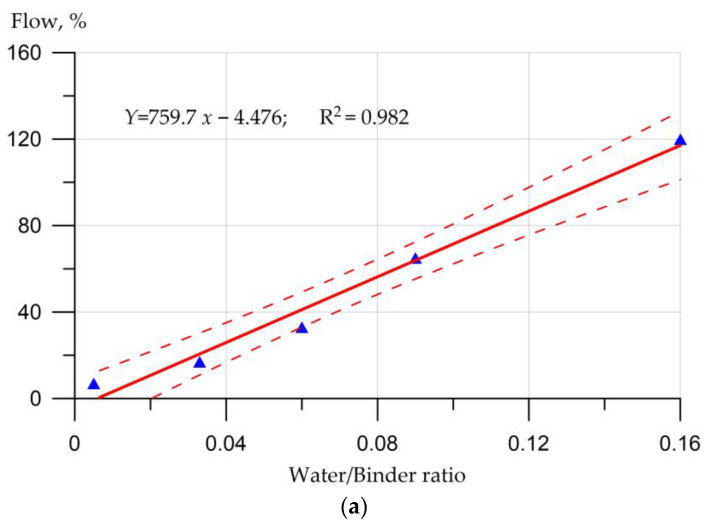

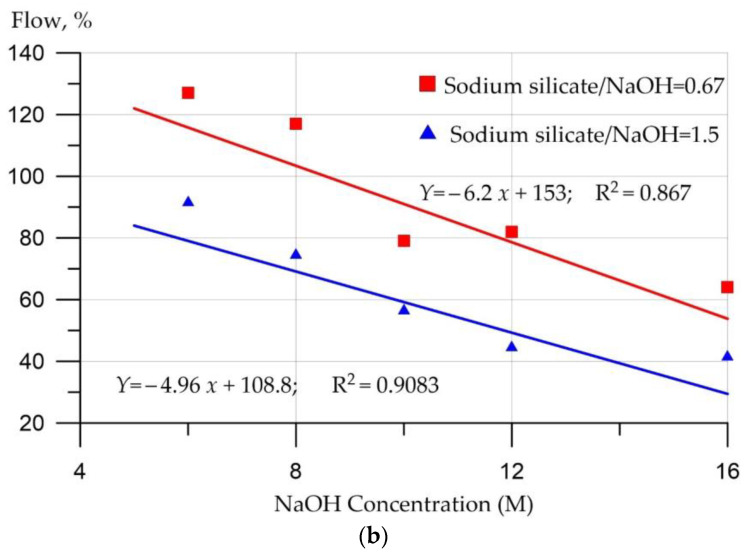
The main factors affecting the workability of geopolymer concretes, and the degree of their influence according to [144]: (**a**) water-binding ratio; (**b**) NaOH concentration.

The workability of geopolymer concretes depends on the concentration of the activator in relation to the main components of the geopolymer composition. It can also be increased by increasing the amount of water in relation to the binder or by adding superplasticizers and hardening retarders. The workability of geopolymer concretes also deteriorated with increasing NaOH concentration [143,144]. Figure 10 shows the influence of the main factors on the workability of geopolymer concretes according to [144].

The hardening time of geopolymer concretes depends on the concentration of NaOH, the proportion of silicates to hydroxides, the composition of the binder, and the content of superplasticizers in the mixture. Increasing the concentration of NaOH reduces the hardening time by accelerating the process of polymerization of geopolymer compositions. However, the short hardening time of the composition significantly worsens its workability [145,146].

Shrinkage during the hardening of geopolymer concrete significantly affects the process of formation of shrinkage cracks in it. Geopolymer concretes have greater shrinkage than OPC based concretes due to the greater number of mesopores than OPC. Geopolymer concretes shrink more than OPC-based concretes due to the fact that the alkali-activated binder itself has much greater shrinkage than OPC, and also due to the greater number of mesopores than OPC. Reducing the number of micropores and shrinkage of geopolymer concrete is possible by selecting the optimal amount of activator, the proportion of silicates to hydroxides and activator to binder, as well as by controlling the hardening conditions. It is also important to note that there is a wide range of different raw materials and types of activators for geopolymer concretes, which under different hardening conditions have quite different structural changes during hardening [147]. The polymerization reaction of geopolymer concretes is a rather complex process, which is significantly influenced by temperature conditions, raw materials and their interaction. The strength of the final hardened material, based on the structure created by the interaction of aluminosilicates with activators depends on how this reaction takes place and the interaction of the components of geopolymer concrete. The main influence on the process of formation of polymeric spatial chains is exerted by the proportion of the main components of the mixture, namely: the proportion of the activator to the binder, silica materials to alumina, silicates to hydroxides, and sodium oxide to silicon dioxide. It is known that the addition of sodium oxide to geopolymer concrete has a positive effect on the polymerization process, the microstructure of the material and its durability. A significant influence of the temperature conditions of hardening of geopolymer concrete on the course of the polymerization reaction was also noted [148]. Curing at room temperature provides greater strength of the hardened material in the later stages of curing with low strength in the initial stages of curing, while curing at elevated temperature provides strength in the early stages of hardening higher than at room temperature; however, the strength in the later stages of curing will be lower. An increased hardening temperature accelerates the course of polymerization reactions; however, at the late stages of hardening, increased evaporation of moisture due to high temperature does not allow silica and alumina to fully dissolve and react, as a result of which shrinkage, porosity, and the number of microcracks increase [146].

The process of polymerization of geopolymer concretes is exothermic and is associated with a significant release of heat in the process of hydration of the mixture components. The polymerization process, which occurs during the interaction of aluminosilicates with alkaline activators, occurs more intensively than the hydration process of OPC, as a result of which the heat released by geopolymer compositions during hardening is higher. The heat released by geopolymer concretes during the hardening process largely depends on the type of activator used and the type of feedstock. It has been established that hydroxide activators significantly increase the temperature released during the hardening process of the geopolymer composition [149]. The type of aluminosilicates also affects the temperature formation during the polymerization reaction of geopolymer compositions. Thus, it has been determined that geopolymer concrete based on metakaolin binder actively releases heat in direct proportion to strength gain, while geopolymer concrete based on FA begins to release heat only after 25 h from the start of the reaction, which is due to the difference in the polymerization processes of these compositions [150].

**Figure 11:**
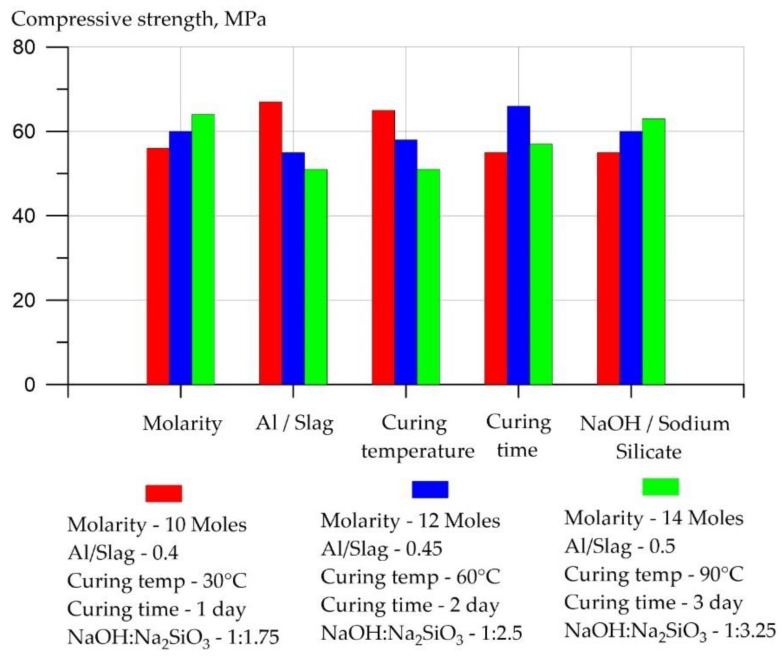
The main factors affecting the CS of geopolymer concretes at the age of 28 days, and the degree of their influence according to [2].

The strength characteristics of hardened and hardened geopolymer concrete are determined by its components and their granulometric composition [151]. The CS of geopolymer concrete depends on the type of sand and the proportion of binder to sand. So, when using lime sand, the strength of geopolymer compositions is reduced compared to conventional quartz sand [152]. The proportion of binder to sand, as a rule, has a positive effect on strength when it reaches its optimum value. It was found that the maximum strength of geopolymer concrete can be obtained with a proportion of binder to sand equal to 0.5. Exceeding this value results in a loss of CS. It was also found that with an increase in the proportion of silicon dioxide to aluminum oxide, the latter actively participates in the polymerization reaction at the early stages of hardening, which leads to its deficiency at the later stages of the polymerization reaction [153]. It has also been found that with an increase in the molarity of the constituents of geopolymer concrete, during the improved polycondensation process, the CS of the hardened material also increases. It was found that with an increase in the molarity of sodium hydroxide and the proportion of sodium metasilicate to sodium hydroxide, an increase in the CS of alkali-activated geopolymer concrete based on GGBS was observed, while an increase in the proportion of alkali activator to GGBS shows an inverse effect on CS [154]. The main factors affecting the CS of geopolymer concretes at the age of 28 days, and the degree of their influence are shown in Figure 11 according to [2]. The ratios of Al/Slag and NaOH/Sodium silicate were provided in terms of mass.

According to [2], it can be noted that with an increase in the molarity of NaOH and the value of the Na_2_SiO_3_/NaOH ratio, the CS of geopolymer concrete based on slag increased, while an increase in the proportion of activator to binder led to a decrease in CS.

The weak point of concrete based on OPC is the low TS relative to the CS. Studies of geopolymer concrete have shown that its TS during thermal hardening is similar to the TS of concrete on OPC and that the dependence of the TS of geopolymer concrete on its CS is similar to that of concrete with OPC as a binder [154]. The TS of geopolymer concretes, as with ordinary concrete with Portland cement as a binder, is determined by axial tensile tests, cube splitting tests, and flexural tensile tests. It was found that the TS of geopolymer concretes during normal hardening increases after the inclusion of 56% nanosilicate in the mixture [155]. It has also been found that the combination of sand and binder has a certain effect on the TS, which, with an increase, reduces the TS of geopolymer concrete. The flexural TS of geopolymer concrete is lower than that of concrete based on OPC due to the lack of calcium oxide in its composition, the addition of which improves the axial and flexural TS of geopolymer concrete [156].

The modulus of elasticity of geopolymer concretes with the same strength is lower than that of concretes based on OPC. Presumably, this is associated with the processes occurring during the polymerization of geopolymer concretes and with their aluminosilicate base [157]. The elasticity modulus of geopolymer concretes is significantly affected by the type of coarse aggregate, the type of binder, temperature and hardening time [158]. Additionally, the modulus of elasticity largely depends on the content of sodium hydroxide in geopolymer concrete, an increase in the amount of which also increases the value of the modulus of elasticity [159].

The main factors that ensure the durability of geopolymer concrete, as well as other building materials, is its ability to withstand atmospheric influences, the chemical effects of aggressive environments, abrasion and other types of wearing effects. The durability of geopolymer concretes is determined by their properties such as sorption capacity, saturated water absorption, approximate volume of open pores, permeability for chloride and sulfate ions, as well as other acids [160]. Compared to concretes based on OPC, geopolymer concretes have significantly longer durability due to the complex polymer structure, which provides not only significant strength characteristics but also a better ability to withstand acidic and mechanical stresses [161]. The type of binder also plays a significant role. For example, slag-based geopolymer concretes have better durability compared to FA-based geopolymer concretes since their geopolymer structure is more stable [162]. However, geopolymer concrete based on FA showed better durability than conventional concrete based on Portland cement [66,163].

Table 3 presents the values of the physical and mechanical properties of geopolymer concrete in accordance with the types of precursors and alkaline activators.

The properties of geopolymer concretes are determined by a number of characteristics of their raw materials [175,176]. These include the molarity of the activator mortar, the proportion of activator to binder, silicates to hydroxides, silica to alumina, and curing temperature [176]. Each of these parameters affects the geopolymer composition at all stages of its hardening [78,84,86,155,177,178].

Despite the variety of binders for geopolymer concretes, many of their characteristics are determined by the activator, in the alkaline environment in which the aluminosilicate polymerization reaction will take place. The main characteristics of the activator are its type and molarity, that is, the concentration of the activating mortar. When interacting with different types of binders, activating mortars can affect the properties of geopolymer concrete in different ways. Thus, an increase in the concentration of the activating sodium hydroxide mortar when mixed with alkali-activated slag increased the CS of the final composition, since an increase in the activity of the alkaline medium led to the formation of a large amount of products involved in the hydration reaction [145]. Increasing the molarity of the activator also increased the CS and worsened the workability of the geopolymer concrete based on FA. Just as in the previous case, the increase in the activity of the alkaline medium of the activator led to the fact that the aluminosilicates in the binder began to dissolve faster, which had a beneficial effect on the process of formation of the spatial polymer structure of geopolymer concrete. However, it was also noted that an increase in the concentration of the activator to more than 35–45% of the total mass of the binder reduces the strength of geopolymer concrete, and this trend continues all the time that the polymerization reaction takes place. This is attributed to the fact that at too high alkali content, precipitation of the aluminosilicate gel occurs [68]. As far as alkaline activators containing soluble silicates are concerned, as compared with mortars based on hydroxides, the reaction with their participation proved to be faster and more active [179]. At the same time, the strength of such geopolymer concretes is higher than that of compositions with an activator based on hydroxides and increases with an increase in the concentration of the activator [180]. Summarizing the above, we can conclude that with an increase in the molarity of the activating mortar, the CS of geopolymer concrete increases.

As mentioned earlier in Section 3, the curing temperature has a significant impact on the strength of geopolymer concretes. The high curing temperature provides an accelerated polymerization reaction and the formation of an aluminosilicate geopolymer structure of the final material, which leads to increased strength in the early stages of curing. However, at the same time, the loss of water in the mixture increases, due to the possible deficiency of which the final strength of the material in the later stages of hardening may be lower compared to geopolymer concrete hardening at normal temperature. It is important to note that increased water losses lead not only to a slowdown in the dissolution of silica and alumina along with their subsequent polymerization but also to an increased formation of pores and microcracks, which also negatively affects the final strength of the material. The curing of geopolymer concretes at room temperature results in the resulting hardened material having greater strength due to a more stable and homogeneous polymer structure, as well as a smaller total pore volume [148]. It should be noted that geopolymer concretes based on GGBS and based on FA have a different composition and an increased curing temperature has a much more positive effect on geopolymer concretes based on FA than based on GGBS. Thus, the strength of geopolymer concretes based on FA at an elevated curing temperature is much higher than at room temperature curing [103].

The proportion of its main components—an activating alkaline mortar and aluminosilicate binder—has a significant impact on the strength of geopolymer concretes. The alkaline environment of the activator is necessary for the dissolution of silica and alumina contained in the binder, which subsequently form a spatial polymer structure that determines the strength properties of the final material. As a rule, an increase in the amount of activator in relation to the binder leads to a proportional decrease in the strength of the final material [2]. Moreover, this trend is typical for geopolymer concretes both based on GGBS and FA. This is due to the insufficient amount of aluminum and silicon particles in the aqueous phase and, as a result, disruption of the process of hydroxylation of the binder particles. A decrease in the amount of the activator in relation to the binder leads to the fact that the mixture loses its workability due to insufficient wetting of the binder particles [181].

Researchers [182] revealed the features of the influence of amorphous and crystalline silica and alumina in the composition of the starting materials for the production of geopolymer concretes on the strength characteristics of the final material. In [52] six compositions were considered with different starting materials to determine the dependence of the CS of geopolymer concrete on the amount of amorphous and crystalline silica and alumina in the raw materials. Compositions based on FA, GGBS and two types of metakaolin were used. The prototypes hardened at high temperatures for 72 h. The age of the prototypes at the time of testing was 7, 14, 28 and 60 days. The results of the experiments and electron microscopy showed a noticeable dependence of the CS of all geopolymer compositions on the presence of amorphous silica and alumina in the starting materials. Crystalline silica, which is part of the raw material, did not enter into the geopolymerization reaction and was also in large quantities in the composition of geopolymer concrete after the formation of its structure. The researchers concluded that the properties of geopolymers can be significantly changed by relatively small changes in Si and Al concentrations during synthesis, where increasing the Si/Al proportion to a certain extent leads to an increase in the CS of the material [52]. In [183], the influence of the calcination temperature of kaolinite clays on the properties of geopolymer binders was considered. Clay fractions of three kaolin minerals were studied. Clay fractions were subjected to chemical and thermal analysis, as well as X-ray diffraction analysis, and, further, calcined at a temperature of 450 to 800 °C. The obtained amorphous material was dissolved in a highly concentrated alkaline solution to obtain a geopolymer composition, whose properties, such as shrinkage, hardening time and CS, were subsequently studied during experiments. The obtained hardened samples of the geopolymer cement paste were also subjected to X-ray diffraction analysis, analysis using electron microscopy and Fourier transform infrared spectroscopy. The hardening time of geopolymer cement pastes produced from clay fractions calcined at 450 °C was very long and amounted to 21 days under laboratory conditions at room temperature. For clay fractions calcined at 500 and 700 °C, the hardening time of geopolymer pastes decreased with increasing temperature and varied in the range from 130 to 40 min. At temperatures above 700 °C, the hardening time began to increase. Shrinkage of the hardened geopolymer paste between 21 and 28 days of age was at its lowest for pastes calcined at 700 °C. When this value is exceeded, the shrinkage value started to increase. The CS of the hardened zeopolymer cement pastes ranged between 11.9 and 36.4 MPa. It increased in samples whose raw material was calcined at a temperature between 500 and 700 °C, but when the latter value was exceeded, it began to decrease. The authors concluded that the optimal temperature for calcining raw materials of geopolymers to improve their mechanical properties is 700 °C [183].

## 4. Geopolymer Concretes with Partial Replacement of Conventional Portland Cement with Aluminosilicate Binders

Despite the outstanding qualities of geopolymer concrete with the complete replacement of OPC with aluminosilicate binders, geopolymer concrete with a partial replacement of Portland cement with aluminosilicates is very popular. The peculiarity of such concrete is that in the process of hydration, an aluminosilicate binder first enters into the reaction, which, when interacting with water, forms a film of positively charged calcium ions, which prevents the further course of the reaction. However, calcium hydroxide, formed during the hydration of the constituents of OPC, raises the pH level, which destroys the calcium ion film [144]. At the same time, calcium hydroxide, interacting with aluminosilicates during the pozzolanic reaction, which is a simple acid reaction between calcium hydroxide Ca(OH)_2_ or CH and silic acid H_4_SiO_4_, forms calcium silicate hydrate. Thus, in the process of hydration of geopolymer concrete with partial replacement of OPC with aluminosilicate binder, hydration of aluminosilicates, hydration of Portland cement and the pozzolanic reaction of calcium hydroxide with aluminosilicates occur [2]. The simultaneous presence of the products of cement hydration and the formation of a polymer structure densifies the final microstructure of geopolymer concrete in the mixture, which has a positive effect on its physical and mechanical properties [144]. At the same time, the hardening time of geopolymer concrete with partial replacement of OPC with aluminosilicate binder is significantly less than that of geopolymer concrete with only aluminosilicate binder, with a slight decrease in workability [21,82]. Summarizing the above, it can be noted that the presence of OPC in geopolymer concrete, along with an aluminosilicate binder, improves the physical and mechanical properties of the final material due to the improvement of its microstructure due to the formation in the process of hydration of a large amount of calcium-rich aluminosilicate gel (calcium aluminosilicate hydrate), which is actively involved in polymerization reactions.

The addition of OPC somewhat improves the microstructure of geopolymer concrete. This is due to the coexistence of hydration products and polycondensation products. Such changes in the microstructure of composites lead to a decrease in water absorption, porosity, sorption and chloride permeability of geopolymer concrete. In general, the addition of OPC makes the microstructure more compact and denser, in which the interfacial bonding of the fibers with the matrix is better [144].

**Figure 12:**
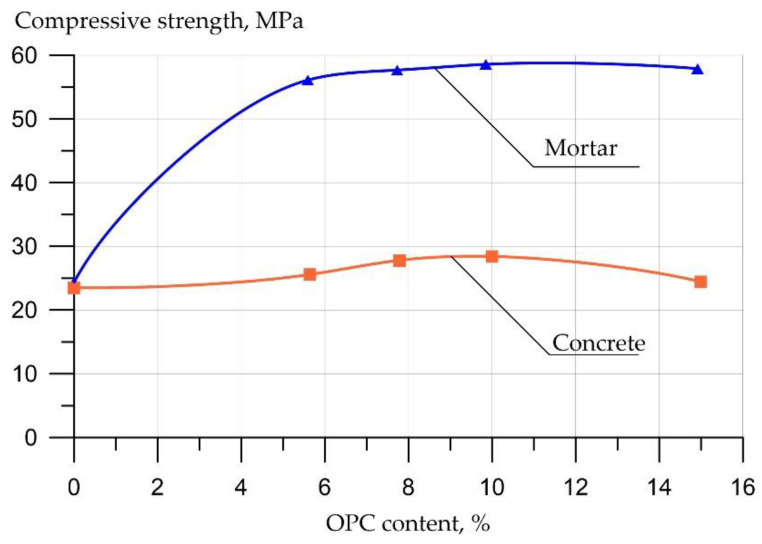
Dependence of the CS of geopolymer concrete based on low-calcium fly ash on the content of OPC (in terms of binder mass) after 28 days of curing [167].

Figure 12 shows the dependence of the CS of geopolymer concrete on the content of OPC according to [167].

According to [167], it was found that the addition of OPC in an amount of 5% increased the CS of geopolymer concrete and mortar by improving their microstructure due to the formation of a large amount of calcium-rich aluminosilicate gel.

## 5. Modern Trends and Innovations in the Field of Research of Geopolymer Concretes

The environmental friendliness of geopolymer concrete compared to concrete on OPC, as well as its outstanding physical and mechanical characteristics, determine the growing popularity of this material in construction. The replacement of concrete based on OPC with geopolymer concrete based on aluminosilicate binders is consistent with the concept of sustainable development. Technologies for the use of geopolymer concretes are being actively explored to reduce energy costs and greenhouse gas emissions. However, geopolymer concrete is a promising material for the construction industry also in terms of its strength properties, outstanding resistance to abrasion, aggressive environments and high temperatures [1]. In recent years, the attention of researchers has been attracted by technologies such as 3D printing using geopolymer concrete, geopolymer concrete based on nanomaterials, self-compacting geopolymer concretes, and determining the impact of the use of geopolymer concretes on global warming. Consider below the most interesting directions from our point of view.

### 5.1. Assessment of the Impact of Geopolymer Concretes on Global Warming

The assessment of the impact of a particular technology on global warming is usually conducted using the GWP (Global Warming Potential) coefficient. It provides an assessment of the degree of impact of various greenhouse gases on global warming over a certain period of time in comparison with the standard, which is taken as carbon dioxide, whose GWP is equal to 1. When assessing the global warming potential of a greenhouse gas, the number of years after the release, during which the gas exists in the atmosphere is calculated. The technology for the production of geopolymer concretes can reduce greenhouse gas emissions by up to 64% compared to the technology for the production of concrete using OPC. However, differences in the impact on greenhouse gas emissions between different types of geopolymer concretes are not so significant [6].

It has now been established that the production of geopolymer concrete has a significantly lower global warming potential than the production of OPC [24]. It has been found that geopolymer concretes, regardless of the type of aluminosilicate binder, have a significantly lower impact on global warming than concrete based on OPC [88]. Despite this, the global warming potential of geopolymer concretes can be further reduced. Thus, it has been established that the environmentally friendly use of sodium hydroxide isolated from sea salt can reduce the global warming potential by up to 64% in comparison with concrete based on OPC [184]. Additionally, an important argument in favor of the use of geopolymer concretes is their better strength properties and durability compared to concrete based on OPC [185]. However, despite the low impact on global warming, geopolymer concretes have other environmental impacts, depending on the type of binder that is used in a particular mixture. This is due to the fact that industrial and agricultural wastes are often used as binders for geopolymer concrete, some of which can have a negative impact on the environment. Most often, this is the pollution of fresh and seawater, eutrophication of water bodies, and changes in natural abiotic factors [186]. Even despite the outstanding resistance of geopolymer concrete to seawater and aggressive environments, the above factors are a strong barrier to the use of geopolymer concrete as building materials for marine and hydraulic structures. A comparison of the influence of various binders on the environment showed that this factor largely depends on the chemical composition of the binder and the amount of silicates. In general, binders that require large amounts of sodium silicate to polymerize have a higher negative impact on the environment and global warming potential [187]. Geopolymer concretes based on FA and GGBS are much safer in terms of global warming potential and CO_2_ emissions than geopolymer concretes based on metakaolin, since metakaolin requires a large amount of silicates for the polymerization reaction [52]. However, geopolymer concrete containing 30% metakaolin in the binder composition has higher strength characteristics than concrete on OPC, and a much lower global warming potential [188]. 

It is important to note that the global warming potential is a very relative factor when it comes to the use of a material such as geopolymer concrete. This relativity is due to the fact that different calculation methods consider different types of activities associated with the use of geopolymer concrete. It was found that when taking into account all activities and energy processes associated with the production and use of geopolymer concrete, from the selection of raw materials to the placement of the finished mixture in the formwork, taking into account the transport of raw materials, the amount of CO_2_ emissions was only 9% less than concrete on OPC [189].

### 5.2. Three-Dimensional Printing Using Geopolymer Concrete

In recent years, 3D printing technology has gained sharp popularity due to the high speed of the production process, and the accuracy of execution, automation and manufacturability; 3D printing in one form or another is used in almost all areas of activity, going beyond global production and firmly entrenched in ordinary everyday tasks. However, the aerospace, automotive, aircraft and construction industries have become pioneers in the use of this technology. In construction, 3D printing technology provides not only the freedom to implement design mortars, but also the automation of construction processes, the reduction in waste, the consumption of raw materials and energy, and the absence of the need for highly paid labor [53]. The above is consistent with the concept of sustainable development, which is in harmony with the advantages in this regard of geopolymer concrete over concrete based on OPC. The combination of 3D printing technology, which is promising from the point of view of the concept of sustainable development, and geopolymer concrete, as an environmentally friendly material, can help reduce environmentally unfavorable factors for the environment and better control over them [190]. From this point of view, the development of this innovative technology is promising and consistent with sustainable development.

3D printing technology using geopolymer concrete implies certain mixture characteristics suitable for automated paving, as well as the use of superplasticizers. A number of studies have been devoted to determining the optimal proportions of mixtures based on various types of binders for use in 3D printing technology. Compositions with a binder based on GGBS, FA and microsilica were studied. The influence of the content of the activator in relation to the binder and the proportion of water to the binder were investigated. The best strength characteristics were obtained at a water-to-binder proportion of 0.33 and an activator amount of 8% of the binder volume [191]. The addition of GGBS to geopolymer concrete based on FA showed an improvement in its microstructure and strength but had almost no effect on the deformability of the material [192]. The addition of microsilica to a similar composition improved the yield strength and viscosity of the final material, which was due to the shape of the binder microparticles and the change in their surface area. Each change in the composition of geopolymer concrete, regarding the type of activator and its amount, the type of binder, and the presence of superplasticizers, leads to a change in the course of the polymerization reaction of geopolymer concretes [193]. However, 3D printing technology imposes additional limitations, since each type of 3D printing machine has its own advantages, limitations and features of operation, and its choice is a unique task for each individual case.

### 5.3. Nano-Modified Geopolymer Concrete

The spatial polymeric aluminosilicate structure of geopolymer concretes provides their outstanding physical and mechanical characteristics compared to concrete based on OPC, which together with the environmental friendliness of geopolymer concretes, makes them one of the main alternatives to traditional concrete [180]. The type of binder has a significant effect on the microstructure of geopolymer concrete. Thus, compositions with a binder based on FA and GGBS have a more stable and denser microstructure, which ensures their better mechanical properties. However, geopolymer concretes with binders based on agricultural waste and clay minerals have a porous microstructure and no outstanding physical and mechanical properties, together with brittleness, high shrinkage, and a high content of microcracks. To improve the properties of such compositions, mineral additives and external agents are included. One such additive is graphene. This material is obtained from graphite by mechanical action. Graphene is a layer of carbon atoms one atom thick interconnected to form a hexagonal two-dimensional crystal lattice [194]. The unique thermal, electrical, mechanical, optical, catalytic and biological properties of graphene make it a special and highly demanded material for high-tech production. Graphene oxide is commonly used as an additive in building cement mortars. Its inclusion in the mixture significantly densifies the microstructure of the final composition, reduces the number of microcracks, reduces brittleness, and thereby significantly increases the strength properties [195]. It was found that the inclusion of graphene oxide in geopolymer concrete with a slag-based binder led to an increase in TS in bending by 20% at a graphene content of 0.01% by weight of the binder [196]. The inclusion of graphene in geopolymer concrete based on FA increased the CS by 2.16 times and the bending TS by 1.44 times. However, the addition of graphene in a volume above the optimal value and an increase in the amount of sodium hydroxide led to a decrease in TS in bending [197]. When adding graphene oxide to geopolymer concrete, not only an improvement in strength properties was observed, but also in deformability, wear resistance and durability. In addition to improving the physical and mechanical properties of geopolymer concretes nanomodification with graphene oxide leads to a decrease in the fluidity and workability of the mixture. This effect can be leveled by adding superplasticizers or microsilica [198].

In recent years, the interest of researchers has been attracted by the effect of nanomodification of geopolymer concretes based on various types of aluminosilicate binders with graphene oxide using 3D printing technology [199].

### 5.4. Monitoring the State of Structures Using Self-Sensitive Geopolymer Concrete

The development of smart materials is one of the main directions for the development of new technologies in the construction industry which allow continuous diagnostics and analysis of the state of building structures without their destruction. With regard to geopolymer concretes, at the moment in scientific publications, topics related to the piezo-resistant behavior of geopolymer concretes under various deformations and the development of technologies that allow using this factor for the continuous monitoring of the state of building structures are gaining popularity. The authors of [200] carried out a work devoted to the study of the piezoresistance behavior of a geopolymer material based on FA with the addition of carbon black during compression. Prototypes were made in the form of cubes with the addition of carbon black in the amount of 0.5%, 1% and 2%, in which four copper electrodes were embedded. To obtain a comprehensive characteristic in the process of increasing the compressive load during the axial compression test, the electrical resistivity, longitudinal deformation and acoustic emission were recorded. The samples were tested in two modes: repeated loading with small compressive forces and continuous loading until failure. The results of the experimental studies carried out showed the presence of piezoresistance for all tested mixtures, but the best self-sensitive properties were obtained with 0.5% carbon black impurities. A comprehensive analysis of the research results showed that the FA-based geopolymer is subjected to residual deformations, and the addition of carbon black changes its character from quasi-brittle to sufficiently ductile. The combination of electrical and acoustic methods makes it possible to control materials far beyond the operating range of the load cell.

The work [201] is devoted to the technology of robotic application of a self-sensitive geopolymer based on metakaolin for monitoring the state of concrete of building structures. The strength of the hardened geopolymer composition, according to the authors, was 20 MPa, and the adhesion to concrete was 0.5 MPa. The geopolymer composition was applied to the concrete base in areas of 250 mm^2^ in laboratory conditions. Four electrodes were installed in each section, measuring the deformation and temperature of the concrete base with an accuracy of 1 μm and 0.2 °C, respectively. The research results showed the possibility of using a robotic method of applying self-sensitive geopolymers for further analysis and monitoring of the concrete state of building structures.

### 5.5. Self-Healing Geopolymer Concrete

In recent years, one of the most interesting areas in the field of building materials is the creation of self-healing materials for building structures. Of particular interest to researchers is the development of technologies for self-healing concrete. For example, researchers [202] considered the issue of creating self-healing bioconcrete using bacteria of the species Bacillus megaterium. Bacteria were added to the mixing water of bioconcrete in the amount of 105 cells per ml of mixing water. After the bioconcrete hardens, the bacteria enter a dormant state, which is interrupted by the formation of a crack, through which microorganisms have access to air and water. As a result of the vital activity of bacteria, the crack is filled with spar crystals. Due to the formation of spores that have a thick wall, Bacillus megaterium bacteria can survive without any damage in the body of concrete for up to 200 years, in anticipation of favorable conditions for life, which significantly exceeds the planned life of most concrete and reinforced concrete structures. According to the results of the research, the authors concluded that the technology of bioconcrete is promising for the creation of self-healing materials. The CS of bioconcrete increased by 19.7% compared to conventional concrete without bacteria. It was also noted that the minimum crack recovery time is 30 days. The authors of the study [203] used two types of bacteria (Megaterium and Subtilis) to create self-healing bioconcrete, which were added in various concentrations (107 and 108 colony-forming units), in various amounts (1, 5, 10 and 15% of the mass of cement) and with different amounts of nutrients (0.1%; 0.6% and 1.2% calcium lactate by weight of cement) into the concrete mixture to determine the effect of these variable factors on the self-healing process of concrete. The resulting material was tested for water permeability, CS and chloride ion permeability. The results of the study showed a significant increase in the CS of concrete, improved water permeability and resistance to the penetration of chloride and sulfate ions. The best performance was achieved with the use of Megaterium bacteria in the amount of 15% by weight of the cement with the addition of calcium lactate in the amount of 1.2% by weight of the cement. The depth of water penetration, in this case, decreased to 56–59% compared to control samples that do not contain bacteria.

By analogy with ordinary concrete, the above technology can also be applied to geopolymer concrete, despite the concentrated alkaline environment in which the geopolymerization reaction takes place. According to [204], some of the bacterial species are spore-forming and resistant to alkali, which allows them to survive for more than 200 years even in dry conditions. At the same time, the technology of bacterial concrete, as applied to geopolymer concrete, remains practically unchanged. When preparing a concrete mix, just as in the case of ordinary concrete, a precursor or nutrient is added to it, which is usually calcium lactate (Ca(C_3_H_5_O_2_)_2_) and various types of bacteria that can simply be added to the mixture or located in special microcapsules that will open when cracks form. When cracks occur, the bacteria come out of their dormant state and, with the help of certain enzymes, produce a precipitate of CaCO_3_, which seals the cracks in the concrete up to a certain opening width. Relatively recent studies [205,206] have been devoted to the application of a biomethod to seal cracks by adding certain types of bacteria to the concrete mixture.

As an alternative method for creating self-healing materials, a number of studies have proposed the use of hollow fibers that are located in the body of the material of construction, by analogy with arteries in a living organism. Hollow fibers may contain several material components, which, when released during interaction with each other or with air, seal the crack initiation site [207,208]. Additionally, by analogy with the previous method, instead of hollow fibers, it is proposed to introduce microcapsules, which, if cracks occur, will be damaged, releasing a reducing agent [209,210].

Although the above methods for creating self-healing materials are applicable to both conventional concrete and geopolymer concretes, the latter are known for significantly higher resistance to high temperatures, which opens up additional possibilities. Thus, researchers [211] proposed adding glass particles and aluminum oxide plates to a geopolymer mixture based on metakaolin. As a result, when the hardened geopolymer composite was exposed to a temperature of 850 °C, the cracks were filled with glass particles. In general, a summary of the results of research on self-healing technologies applicable to geopolymer concretes can be summarized in Table 4.

Geopolymer concrete, as mentioned earlier, is more environmentally friendly than conventional concrete due to its low carbon footprint. Self-healing concrete technologies can enhance these properties of geopolymer concrete, reducing environmental pollution by increasing the service life of geopolymer concrete structures, reducing repair costs and saving energy.

## 6. Discussion

In order to fully evaluate this review in terms of scientific novelty and practical significance, it is necessary to conduct a comparative analysis between the study and reviews previously performed by other authors. It is conditionally possible to divide the studies conducted by other authors, grouping them according to a number of factors. For example, part of the research was devoted to the scientometric analysis of the development of research on geopolymers, and their economic and environmental prospects [1,32,52], without paying much attention to the properties of the materials themselves. Many authors have worked on a review of studies devoted only to the microstructure and properties of geopolymer concretes [26,28,37,38,39,50,51]. Studies [31,44] were aimed at reviewing technologies for the use of certain types of construction and household waste as components of geopolymer concretes. The authors of studies [33,34] reviewed the methods for selecting the composition of geopolymer concretes, focusing mainly on the methodological component. Studies [40,41,49] have been reviewed within geopolymer concretes using only fly ash as an aluminosilicate binder, not covering other types of binders. It is also possible to distinguish a group of studies devoted to a review of works limited to a rather narrow topic, for example, studies devoted only to activators or 3D printing using geopolymer concrete [22,43,47,48,53,205].

However, within the framework of such a wide and diverse topic as geopolymer concretes, for the most objective assessment of the further prospects for their development, comprehensive review studies are needed to consider promising areas in the study of these materials, taking into account their properties, features and history of research topics. Thus, in the presented work, the review of the development of research on geopolymer concretes is supported by a review of research on the microstructure and properties of these materials, their determining factors and various combinations of geopolymer mixture components. Such a framework made it possible to fully present an overview of research on current trends and innovations in the field of geopolymer concretes, which are presented not as a separate narrow topic, but as part of a global picture of research on these materials.

Prospects for further research are seen in obtaining new knowledge and developing theoretical ideas about the most relevant and priority areas for the use of geopolymer concretes.

## 7. Conclusions

The literature review presented in this article covers a wide range of research in the field of geopolymer concretes carried out in recent years. Based on the information provided, the following main conclusions can be drawn:

(1) Geopolymer concrete is a suitable, environmentally friendly and sustainable alternative to concrete based on OPC with higher strength, physical-mechanical and deformation properties due to its more stable and denser aluminosilicate spatial microstructure. With the active use of agricultural and industrial waste, the production of geopolymer concrete can also become more economical than the production of OPC.
(2) The main factors influencing the properties of fresh and hardened geopolymer concrete mixture are identified and visually presented. The physical and mechanical properties and durability of geopolymer concretes depend on the composition of the mixture and the proportions of its components. The selection of compositions must necessarily consider the main ratios of the components that determine the physical and mechanical properties of the finished material. These include activator-to-binder ratio, silicates to hydroxides, binder type, activator concentration, aggregate fineness modulus, presence and amount of superplasticizers, water-to-binder ratio, and binder-to-sand ratio.
(3) Despite a large number of types of aluminosilicate binders, most researchers have considered geopolymer concretes based on FA and GGBS because of their higher strength characteristics and outstanding physical and mechanical properties compared to other types of binders. Microsilica, metakaolin, perfluorooctanoic acid, high calcium ash and rice straw ash have been considered by researchers as independent binders much less often, but their influence as additives has often been analyzed. The most popular activator mortars used by researchers are sodium hydroxide and sodium metasilicate, as well as their combinations in various ratios. Moreover, sodium metasilicate showed a faster course of the polymerization reaction compared to sodium hydroxide. The course of the polymerization reaction, in addition to the type of binder used, is greatly influenced by the characteristics of the activator, such as its concentration, amount in relation to the binder and reactivity. The optimal value of the proportion of activator to binder allows you to get the best mechanical properties and durability.
(4) Geopolymer concretes with partial replacement of OPC with aluminosilicate binder have a denser and more compact microstructure due to the formation of a large amount of calcium silicate hydrate, which provides improved CS, durability, less shrinkage, porosity and water absorption.
(5) The first studies devoted to the subject of geopolymer concretes were aimed at obtaining new data on the influence of the ratios of the mixture components on the strength and physical-mechanical properties of the final material. They were based on the influence of such factors as the proportion of NaOH/Na_2_SiO_3_, the proportion of activator to binder, the proportion of NaOH to slag, the combined use of various activating compositions, various variations in conditions and curing temperatures, the concentration of activators, various types of coarse filler, the addition of nanomodifiers, the percentage of reinforcement, adding various additives, fibers and coarse aggregates from recycled waste to find the optimal mixture parameters and a combination of various factors to obtain the best properties of the final material. Recent research uses new ideas and technologies, such as 3D printing of fiber-reinforced geopolymer concrete, and needs to be further developed in studies based on the analysis of the properties of compositions obtained with various types of binders and activators.
(6) The technologies of the combined selection of the composition of geopolymer concrete, production of nanomodified geopolymer concrete, 3D printing of building structures from geopolymer concrete, and monitoring the state of structures using self-sensitive geopolymer concrete are considered.
(7) An assessment was made of the potential reduction in greenhouse gas emissions from the production of geopolymer concrete compared to the production of OPC.
(8) The inclusion of nanomodifiers, such as graphene oxide, in the composition of geopolymer concretes reduces the hardening time of the composition and increases the stability of the microstructure of the material, improving its physical and mechanical properties and positively affecting durability.
(9) The most promising areas for further research of geopolymer concretes are the search for new types of aluminosilicate binders and activators; the analysis of the economic and environmental efficiency of geopolymer concretes; the development of methods for the optimal selection of compositions; the analysis of the effect of nanomodification of the composition of geopolymer concrete on the characteristics of the finished material; the search for new technologies that allow more efficient and productive 3D printing using geopolymer concretes; the search for new technologies to improve the properties of geopolymer concretes by nanomodifying the composition with graphene oxide and the search for alternative, more effective nanomodifiers; the search for technologies that can reduce the toxicity of certain types of geopolymer concretes for fresh reservoirs and humans; the search for new technologies and replacement methods concrete on OPC with geopolymer concrete and the search for new methods for monitoring the state of structures using self-sensing concrete. Self-healing concrete technologies can enhance the properties of geopolymer concrete, reduce environmental pollution by increasing the service life of geopolymer concrete structures, and reduce repair costs and save energy.

As a result, a review of studies on modern trends and innovations in the field of geopolymer concretes was made, which was presented not as a separate narrow topic, but as part of a global picture of research on these materials. Prospects for further research are seen in obtaining new knowledge and developing theoretical ideas about the most relevant and priority areas for the use of geopolymer concretes.

## Figures and Tables

**Table 1:** An overview of the altered sorts of aluminosilicate components with a comparison of their chemical composition.

Ref. Number	Name of Aluminosilicate Component	SiO_2_	Al_2_O_3_	Fe_2_O_3_	CaO	TiO_2_	K_2_O	MgO	Na_2_O	Loss on Ignition
[64]	Fly ash	55.90	28.10	6.97	3.84	2.21	1.55	-	-	1.20
[65]		51.11	25.56	12.48	4.30	1.32	0.70	-	-	0.57
[66]		73.50	22.50	1.10	0.40	1.40	0.30	0.40	0.20	
[67]		44.83	29.23	4.66	4.47	-	0.68	1.62	1.32	-
[68]		62.19	27.15	3.23	1.97	1.06	0.89	0.40	0.30	1.75
[69]		55.00	26.00	10.17	2.09	-	1.65	0.80	0.40	3.89
[70]		49.10	34.80	4.50	4.90	-	1.30	0.40	0.40	2.30
[71]		51.80	26.40	13.20	1.61		0.68	1.17	0.31	0.50
[72]		52.40	18.09	0.42	0.33	4.33	0.19	0.02	0.03	20.59
		27.35	50.85	1.88	5.41	2.57	0.35	0.02	0.05	7.74
[73]	Metakaolin	51.35	44.24	0.98	0.13	0.90	0.08	-	-	0.72
[74]		59.70	34.10	0.90			0.10			1.00
[71]		52.10	41.00	4.30	0.09		0.62	1.36	0.01	0.50
[75]		48.88	43.39	3.77	0.98	2.45	0.14			0.35
[76]		52.80	43.70	0.60		0.50	1.20	0.20		
[77]		54.00	47.00	0.40	0.10		0.10		0.30	
[78]		55.01	40.94	0.55	0.14	0.55	0.60	0.34	0.09	1.54
[79]		53.18	42.72	0.97	0.28		0.41	1.58	0.09	0.34
[80]	Rice husk ash	96.03	0.01	0.13	0.53		1.67			1.45
[81]		86.49	0.01	0.91	0.50		2.70	0.13	0.05	8.83
[82]		91.60	0.09	0.64	1.38		5.14			5.43
[83]		90.11	1.19	0.85	0.89		3.84	0.90		4.05
[84]		88.90	2.50	2.19	0.22					4.01
[85]		83.62	3.01	1.63	2.63		4.59	0.96		
[86]		90.13	0.42	0.52	1.23		1.51	0.89	0.51	2.08
[87]		91.70	0.22	0.12	1.01		2.37	0.36	0.13	3.93
[88]		93.30		0.58	1.82		0.88	0.28	0.19	2.25
[89]		86.20	0.46	0.43	1.10		4.60	0.77		4.60
[90]		83.10	2.15	1.10	4.70		2.96	1.50	0.10	1.13
[91]	Ground granulated blast-furnace slag	33.78	13.97	1.44	42.85		0.40			
[66]		30.30	12.90	0.40	47.80	47.80	0.30	4.50	0.40	
[70]		32.60	16.40	0.40	38.70		0.30	7.10	0.30	0.50
[92]		32.70	8.30	43.80	3.70	-	-	0.40	-	-
[63]		33.40	16.90		33.30	0.61	0.16	7.00	2.00	
[93]		34.70	14.40	0.80	42.00			6.90		1.10
[94]		36.00	13.80	0.30	42.60	0.80	0.27	5.80	0.21	0.56
[95]		36.00	11.80	0.30	42.60		0.30		0.20	
[96]		42.47	35.17	13.93	0.58		0.46	4.12	0.15	0.18
[97]		34.38	12.98	1.29	37.33		0.82	5.59	0.29	4.31

**Table 2:** List of alkaline activators with their various characteristics.

Ref. Number	Used Activator	Molarity of Hydroxide	The Proportion of Activator and Binder by Weight
[98]	Na_2_SiO_3_ + NaOH	10 M	-
[99]	Na_2_SiO_3_ + NaOH	10 M	0.6
[100]	Na_2_SiO_3_ + NaOH	12 M	0.45
[63]	NaOH	8 M	-
[101]	Na_2_SiO_3_ + NaOH	6–12 M	-
[102]	Na_2_SiO_3_ + NaOH	8 M	-
[103]	Na_2_SiO_3_ + NaOH	14 M	-
[104]	Na_2_SiO_3_ + NaOH	10–14 M	-
[105]	Na_2_SiO_3_ + NaOH	12 M	-
[106]	Na_2_SiO_3_ + NaOH	12 M	0.5
[107]	NaOH + KOH	4–16 M	0.5
[108]	Na_2_SiO_3_ + NaOH	12 M	0.429–1.0
[109]	Na_2_SiO_3_ + NaOH	8–10 M	

**Table 3:** Properties of geopolymer concrete with various precursors according to [4,164,165,166,167,168,169,170,171,172,173,174].

Ref. Number	Precursor	Alcaline Activator	Workability (mm)	Curing Temperature	Initial Setting Time (min)	Final Setting Time (min)	Compressive Strength (MPa)	Tensile Strength (MPa)	Flexural Strength (MPa)
[164]	Fly Ash	NaOH + Na_2_SiO_3_	710	60–90 °C	-	-	47.54–53.99	-	-
[165]	Fly Ash	NaOH + Na_2_SiO_3_	110–135	75 °C	-	-	10–65	-	-
[166]	Fly Ash	NaOH + Na_2_SiO_3_	240	-	405	570	47.21	-	-
[167]	Fly Ash	NaOH + Na_2_SiO_3_	-	Ambient temperature	66–112	160–245	40	-	-
[168]	Fly Ash	NaOH + Na_2_SiO_3_	-	80 °C	-	-	48	-	-
[169]	Fly Ash	NaOH + Na_2_SiO_3_	-	Ambient temperature	-	-	11.8–29.2	-	-
[4]	Fly Ash	NaOH + KOH + Na_2_SiO_3_	-	-	-	-	24.96–30.11	3.72–4.95	5.22–6.03
[170]	Fly Ash + Slag	NaOH + Na_2_SiO_3_	-	Ambient temperature	-	-	30.5–80.5	8.35	17.95
[171]	Fly Ash + Slag + Palm oil fuel ash	NaOH + Na_2_SiO_3_	145–160	65 °C	-	-	66	-	7.7
[172]	Fly Ash + Slag + High calcium wood ash	NaOH + Na_2_SiO_3_	-	-	20–280	90–360	36.56	-	-
[173]	Fly Ash + Slag + Portland cement + Calcium hydroxide	NaOH + Na_2_SiO_3_	-	Ambient temperature	110–607	110–607	26–58	-	-
[174]	Fly Ash + Slag + Nano silica	NaOH + Na_2_SiO_3_	-	Ambient temperature	-	-	40.28–56.7	-	-

**Table 4:** Results of research on self-healing technologies applicable to geopolymer concrete [202,203,206,207,208,209,210,211].

Ref. Number	Healing Agent	Addition by Weight of Cement, %	Self-Healing Performance
[202]	Bacterial spores	2	Compressive strength restoration (19.7%); cracks filling
[203]	Bacterial spores	15	Depth of water penetration decrease (59%); cracks filling
[207]	Hollow fibers	2	Compressive strength restoration (33.1%); cracks filling
[209]	Microcapsules	4	Compressive strength restoration (60%); cracks filling;
[211]	High temperature activated agents	50	Cracks filling

## Data Availability

The study did not report any data.

## Nomenclature

CO_2_	carbon dioxide
Si	silicon
O	oxygen
Al	aluminum
SiO_2_	silica
Al_2_O_3_	aluminum oxide
Fe_2_O_3_	iron(III) oxide
CaO	calcium oxide
TiO_2_	titanium dioxide
K_2_O	potassium oxide
MgO	magnesium oxide
Na_2_O	sodium oxide
Na_2_SiO_3_	sodium metasilicate
NaOH	sodium hydroxide
KOH	potassium hydroxide
H_2_O	hydrogen oxide (water)
CaCO_3_	calcium carbonate
Na	sodium
K	potassium
Ca	calcium
H	hydrogen
Ca(OH)_2_	calcium hydroxide
H_4_SiO_4_	orthosilicic acid
Ca(C_3_H_5_O_2_)_2_	calcium salt of propionic acid
